# The Historical Development of *Deqi* Concept from Classics of Traditional Chinese Medicine to Modern Research: Exploitation of the Connotation of *Deqi* in Chinese Medicine

**DOI:** 10.1155/2013/639302

**Published:** 2013-11-05

**Authors:** Hong-Wen Yuan, Liang-Xiao Ma, Dan-Dan Qi, Peng Zhang, Chun-Hua Li, Jiang Zhu

**Affiliations:** ^1^School of Acupuncture Moxibustion and Tuina, Beijing University of Chinese Medicine, Beijing 100029, China; ^2^School of Traditional Chinese Medicine, Capital Medical University, Beijing 100069, China; ^3^The Key Unit of Evaluation of Characteristic Acupuncture Therapy, State Administration of Traditional Chinese Medicine, Beijing 100029, China; ^4^Beijing Electric Power Hospital, Capital Medical University, Beijing 100073, China

## Abstract

Although it is difficult in fully clarifying its mechanisms and effects, *Deqi* still can be considered as an instant “sign” of acupuncture response of the patient and acupuncturist, which has a significant value in clinic and research. This paper aims to take a history trace to the development of *Deqi* theory, understand the connotation of *Deqi* based on Chinese medicine theory, and establish an evaluation methodology accordingly. We believe that *Deqi* is not only the needling sensation, but also the perception of changes of *qi* ^′^ flowing of the patient elicited by needling on acupoints. The signs of *Deqi* include the patient's subjective perception (needling sensation), the objective physiological changes (common referred to the skin redness around the acupoints and the response of brain), and the acupuncturists' perception. Although *Deqi* is essential for attaining the effect, it may not be the necessary sign of the ideal efficacy. It is found that the characteristics of *Deqi* sensations, *Deqi*'s intensity, time duration, and the propagation will all affect the efficacy. Thus, acupuncturists should pay attention to elicit and control *Deqi* state, which is also the key point in modern research on the therapeutic implications of *Deqi*.

## 1. Introduction

There is so far little evidence of investigating the relationship between the therapeutic efficacy of acupuncture and the *Deqi* experience. However, as the specific perception of acupuncture stimulation, *Deqi *may influence the physical and psychological condition of the patients. 

The terms of “*Deqi*” and “*Qizhi*” were first found in “*Huang Di Neijing*” [1]. The sentence “acupuncture therapy does not take effect until the arrival of energy,” which is thought to be the basic principle to guide the clinical practice of acupuncture. At present, most acupuncture doctors and researchers in China [2–5] believe that *Deqi* is essential for the efficacy of acupuncture and it is necessary to induce *Deqi* sensation to a certain degree. 

Some clinical results confirm that *Deqi* reveals some connection with the efficacy of acupuncture treatment [6–11]; however, others concluded that *Deqi *sensations did not result in effectiveness [12, 13]. Learning from these studies, we found that studies conducted in China usually applied deep needling (1 to 2 cm) with manipulations every 5 min during needle retention (30 min as usual), so as to maintain certain intensity and time duration of *Deqi* sensations. While the studies abroad mostly insert superficially or manipulate needle only once in the treatment. 

There is one sentence in *Neijing* that talked about *Deqi*: “The *qi* in acupoints is delicate, means that to elicit responses of *qi* (*Deqi*), maintaining *qi*, and watch the movements of energy is an important step in acupuncture therapy” (from *Ling Shu* Chapter 3 on minute needle). It confirmed that “maintaining *qi*” actually refers to the fact that maintaining the intensity and time duration of needling sensation is necessary after *Deqi*. The manipulation every 5 min during needle retention after *Deqi *may be the key to a higher efficacy in the study. Some researchers also pay attention to the dose-effect relationship of acupuncture and consider that the efficacy of acupuncture treatment depends on complex factors, such as the number of needles, depth of needling, point location, needling retention, and interval between two sessions [14]. Therefore, the intensity of needling in most present acupuncture studies might not be enough for achieving therapeutic effects. It may help some confounders to have biased findings toward a negative outcome [15, 16]. These views support that *Deqi* is a key to the treatment efficacy. However, the diversity of perspectives on the relationship between *Deqi* and effect in current studies may be caused by different understanding of *Deqi*. 

The distinct difference in the understanding of *Deqi* can be found in the literatures in China and other countries. Among the *Deqi* related literatures published in China since 1950, there are 137 articles (67%) discussed on the understanding of *Deqi.* There are a variety of definitions of *Deqi* across different textbooks.

## 2. The Definitions of *Deqi* Nowadays

In the latest “Eleventh Five-Year” national plan textbook “Acupuncture and Moxibustion,” *Zhen Jiu Xue* [17] states: “*Deqi*, normally called *Qizhi* in ancient, or needling sensation in modern, refers to the response of channel *qi* elicited by acupuncture stimulation such as lifting, thrusting, as well as rotating the needle after inserting the needle into an acupoint.” The signs of *Deqi* include two aspects, one is the patients' needling sensations, and the other is acupuncturist's perceptions. When obtaining *qi*, the patient may feel sensations of *suan* (soreness), *ma* (numbness), *zhang* (fullness/distention), and *zhong* (heaviness) and sometimes a feeling of heat, cold, pain, itching, muscular twitching, formication, and so forth, and those sensations can spread in some certain directions. A few patients may show the reaction of twitching or involuntary movement of skin and muscles along the stimulated channel, or red skin rash, or red or white lines on the skin around the punctured area. At the same time, the acupuncturist may feel heavy, tight, or vibration of the needle. If *Deqi* is not evoked, the patient would not have any special feeling or reactions, and the acupuncturist may feel the needles are loose and empty.

The widely accepted definition of *Deqi* in English language textbooks is: “Traditional acupuncture involves stimulation with very fine needles inserted into defined sites on the body, eliciting a composite of sensations, termed *Deqi*, which is considered to be related to clinical efficacy in traditional Chinese medicine” [18].

The latest Chinese-English Dictionary of Traditional Chinese Medicine (TCM) defines *Deqi* as “needling sensation, which refers to the patient's response to sore, numb, distention, electric shock and the doctor's heavy and tight sensation coming from beneath the needle” [19]. 

Several randomized controlled clinical trials in a large sample with a significant influence usually defined *Deqi* as “an irradiating feeling deemed to indicate effective needling” [6, 20, 21]. 

Recently, the most common definition of *Deqi* is “the needling sensations of both patient and acupuncturist.” However, in the theory of TCM acupuncture, needling sensation is the most significant manifestation of *Deqi*, but they are not equal on the level of connotation. In addition, modern functional brain imaging technology also demonstrated that classic *Deqi* sensation, such as a sense of soreness, had different effect on the certain area of the brain compared with sharp pain [22] which further supports the point that *Deqi* is not equal to needling sensation.

## 3. The Origin of *Deqi* Theory

The theory of TCM acupuncture generated from traditional Chinese culture. Therefore, prior to the study of *Deqi* theory, we should firstly understand its background, and then the connotation of *Deqi* could be explained by modern research methods.

*Deqi* is derived from the concept of “*Qi*” in “*Huang Di Neijing*” (written in AD 206~221 [23]). “*Qi*” is an important concept in *Neijing* to describe the activities of human life. The ability to maintain a healthy state is normally called healthy *qi* (*zheng qi*) or grain *qi* (*gu qi*), while the exogenous pathogenic factors that lead to varieties of diseases or pathological changes in the body are called pathogenic *qi* (*xie qi*). 

In *Neijing*, the formation of the theory of “*Qi*” is mainly originated from “the theory of *qi* transformation in life” in Taoism. In the Taoism work “*Huai Nan Zi*” [24], “body”, “*qi*”, and “mind” are composed of three treasures of life. They are interdependent and mutually related. The efficacy of acupuncture would be closely related to the patient's mental status and psychological factors, showing correspondence between “*qi*” and “mind”. Both the patient and acupuncturist will perceive certain sensations after *Deqi*, which is actually the interactions between “*qi*” and “body”. Moreover, TCM holds that the *qi* of the human body corresponds to the seasons, resulting in the relationship between physiological changes and the replacement of the four seasons. Thus, the seasons may affect the difficulty of *Deqi*. For example, the blood flows superficially in summer, during which *Deqi* is easier, while the blood flows deeply in winter and *Deqi* is relatively difficult. In *Neijing*, “*qi*” can also be classified into three categories: organ's* qi* (*Zangqi*), channel's *qi* (*Jingqi*), and vessel's *qi* (*Maiqi*). These three types of *qi* circulate in the body nonstoppingly. Therefore, stimulating acupoints on channel can regulate the certain internal organ to cure the disease. Detecting the changes of pulse can also be applied to determine “*Deqi*” in *Neijing*. 

In summary, the theory of “*Qi*” in *Neijing* is the basis of the traditional acupuncture theory of “*Deqi*.” Phenomenon and impact of* Deqi* are all developed on this basis. TCM acupuncture emphasizes that the acupuncturist should concentrate on the needling process and carefully perceive the sensations below the needle to judge and regulate *qi* of the patient.

## 4. The Development of Connotation of *Deqi*

The view that acupuncture treatment efficacy is closely related to *Deqi* originated in *Neijing.* “If insertion of needle fails to bring about the response of energy, treatment should be continued for as long as necessary. Acupuncture therapy does not take effect until arrival of energy (*Qizhi*).” (from *Lingshu* Chapter 1 Nine needles and twelve original points). What is more, there is a detailed explanation of “*Qizhi*” in *Neijing,* “the principles of needling dictate that needling should stop as soon as energy is brought into harmony, due to tone up the body energy of *yin* and sedate the pathogen of *yang*, or to tone up the body energy of *yang* and sedate the pathogen of *yin*. … The assertion that needling takes effect so long as *Qizhi* means that an excessive disease could be sedated and the deficient energy may be complement.” (from *Lingshu* Chapter 9 From beginning to end). It expresses that the function of acupuncture is “regulating *qi*,” which can be achieved by supplementing and draining acupuncture manipulation. Therefore, pathogens can be eliminated, deficient healthy *qi* can be supplemented, or reversed flow of *qi* be adjusted, leading to a state of *yin-yang* balance. The connotation of “*qi*” in “*Qizhi*” refers to healthy *qi* or grain *qi*. Obviously, *Qizhi* indicates “proper intensity of stimulation” and also the sign of removing the needle. In *Neijing*, “*Deqi*” and “*Qizhi*” are usually considered as the same, regarded as a sign of efficacy [25, 26].

The Difficult Classic (*Nanjing) *[27] written in AD 106~210 [28] normally has similar reputation as *Neijing *in Chinese medicine. The chapter of Seventy-eight Difficult Issues states, “Insert a needle along the route of the channel to induce *Deqi* firstly. Then thrusting the needle inward is a supplementary method, while lifting the needle outward is a draining method.” That text shows that certain needling techniques for supplementary or draining are applied after *Deqi*. *Nanjing* describes* Deqi* as the basis and premise of acupuncture manipulation. It may indicate that *Deqi* and *Qizhi *are not the same. *Deqi* refers to the first period of *Qizhi*, which is the sign of ideal efficacy.

The development of acupuncture can be traced to the *Jin* and *Yuan* Dynasty (AD 1115~1368), clarifying that *Deqi* is the base of acupuncture manipulation. There is one sentence in *Song to Elucidate Mysteries *(*Biao You Fu*) written by *Dou Hanqing starting*, “Then the acupuncturist should perceive *qi* carefully. The sensation of loose and empty beneath the needle means *qi* does not arrive. While heaviness, tightness and fullness sensations suggest that *qi *has arrived. When *qi* arrives, manipulate the needle properly according to cold or heat syndromes; when *qi* does not arrive, wait for *qi* according to deficiency or excess conditions.” It keeps the same point as the *Nanjing*. Till that time, it is found that *Deqi* not only is related to the treatment efficacy, but also can be used to determine the prognosis of the disease. As written in *Song to elucidate mysteries*, “the more quickly of* qi* arrives, the easier the disease is to cure; supposing the *qi* does not arrive, the patient may be hard to cure,” which means that the degree of difficulties of getting *Deqi* can predict the efficacy. Of course, here, the efficacy does not refer to the real-time acupuncture effect but to the significant treatment efficacy of the disease. 

As the acupuncture developed in the *Ming* and *Qing* dynasties (1368~1911), there is a further illustration of the relationship between the acupuncturist's perception of the needle and efficacy. One of the most influenced publications at that time, *The Complete Compendium of Acupuncture and Moxibustion *(*Zhen Jiu Da Cheng*) [29], states, “How to remove the needle depends on the acupuncturist's perception on the needle. Extremely tense and firm sensation beneath the needle indicates the needle is being grabbed by pathogens, not by the healthy *qi*, therefore, the needle cannot be removed. If the needle was removed at this moment, the disease might be palindromia. Instead, needling techniques for supplementary and draining should be applied and retain the needle for a certain period. Only when a looser sensation beneath the needle is felt, the needle can be removed.” According to these texts, acupuncturist should clarify the sensations beneath the needle after *Deqi*. It is called “distinguishing *qi*.” 

Originating from *Nanjing*, the connotation of “*Deqi*” and “*Qizhi*” are different; “*Qizhi*” is obtaining the effect of the final state through the returning of *qi* in human body by normal acupuncture treatment, while “*Deqi*” is the early part of “*Qizhi*,” which means that the *qi* has been elicited to the needle. After realizing *Deqi*, it is still needed to distinguish the character of *qi* and apply the related manipulation or retain the needle, so as to reach the “*Qizhi*” state [30–33].

## 5. The Evaluation Method of *Deqi* Based on Its TCM Connotation

### 5.1. Both Notice the Perception of Patient and Acupuncturist

Evaluation of *Deqi* in ancient Chinese medicine mainly focused on the acupuncturist's perceptions, rather than the patient's needling sensation during an acupuncture treatment. Ancient Chinese acupuncturists believe that during acupuncture, the acupuncturist should concentrate on the changes of *qi *after the needle being inserted, so as to know the situation of patient and disease, then give appropriate needling manipulations to regulate *qi*. Moreover, the ability of “eliciting *qi*,” “distinguishing *qi,*” and “regulating *qi*” reflects the level of healing skill of the acupuncturist. In modern time, *Deqi* is defined as the needling sensation, so the quantitative evaluations of *Deqi *are mainly based on the patient's needling sensations [34–37]. Although the needling sensations of the patient are more direct and sensitive, we suggest that the perceptions of the acupuncturist should be noticed as well, which is the key of “regulating *qi* with the needle.” 

### 5.2. Distinguish the Sensation of Penetrating Skin from *Deqi*

 In TCM, *Deqi *is the sign of the change of *qi*, so it should be elicited when the needle is inserted into the acupoint. Park et al. [38] also find a strong connection between acupuncture sensation and tissue depth; the frequency of prinking and sharp sensation was significantly greater in shallower tissue levels, and the frequency of sensations described as traditional *Deqi *sensation, such as dull, heavy, and spreading, was significantly greater in deeper tissue levels. Therefore, the pain or other sensations which could be felt when the needle penetrated skin is not *Deqi *sensation. In recent needle sensation questionnaire/scale, the item “sharp pain,” “pricking,” and “penetrating” may evaluate the sensation of penetrating skin but not* Deqi.* It would be better to design an instrument for calling attention to the patient to distinguish the sensation of needling penetrating from *Deqi.*

### 5.3. Evaluating *Deqi* More Comprehensively Than Existence and Intensity

*Deqi* is closely related to the treatment efficacy in *Neijing*; however, the formation of *Deqi* is only the first step of achieving the ideal efficacy in traditional theory. Through diagnosing based on *qi *after *Deqi*, retaining the needle skills further improve the acupuncture efficacy. After immediately finishing the treatment after *Deqi*, the patient condition would deteriorate as the doss of stimulation is not enough. It was also stated that “*Qi* extending affected treatment partially” as mentioned in *Zhen Jiu Da Cheng*, which means that the correct direction and a certain distance of *Deqi *spreading can enhance the efficacy. Therefore, the evaluation of *Deqi* existence and intensity is only two aspects in evaluating the relationship between the *Deqi* and the treatment efficacy. The nature of *qi*, intensity, duration, and the spreading should be recorded comprehensively by *Deqi *evaluation instrument, which may influence the efficacy in clinical trials.

### 5.4. Notice the Influence of *Deqi* in “Mind”

The traditional theory of *Deqi* does not merely refer to the needle feeling, but also includes the reaction of “*qi*” in the human body. According to TCM theory, this phenomenon can affect constituting the other two significant elements of the human body: the “body” and “mind;” manifested in “body” is the needle feeling, while that manifested in “mind” referred to the change of the state of brain function, which are the two most important factors in evaluating the *Deqi.*

The study of brain central mechanisms of acupuncture carried out recently is combined with a large number of brain imaging technology (PET, SPECT, fMRI, etc.), which provides a scientific method in investigating the *Deqi* effect. Researchers hope to define a model of *Deqi* brain function by applying the brain functional imaging technology, through which the cases fit this model can be defined as *Deqi*, so it may become easy to distinguish whether to get *Deqi* or not [39]. Kong et al. [40] have confirmed that the signals of fMRI can correctly reflect the specificity *Deqi *state between the different individuals, which provides a reliable method to evaluate *Deqi*.

## 6. Conclusion

Acupuncture is one of the important methods to realize “mediating meridians, regulating the *qi* and blood” in TCM, in which *Deqi* plays an important role in the process of acupuncture. *Neijing* lays the theoretical basis of the theory of *Deqi*. *Deqi* does not only refer to needling sensations, but also involves the changes of *qi* induced by needle insertion into the acupoint. The signs of *Deqi* include the patient's sensations (needling sensations), objective physiological changes (commonly refer to the skin redness around the acupoint as well as the response of brain), and the acupuncturist's perceptions. *Deqi *is closely related to the treatment efficacy; however, it is not a necessary sign for the most ideal efficacy. The characteristics of* Deqi,* including what kind of sensations of both patient and acupuncturist, their prevalence, intensity, time duration, and the propagated sensation along channel will all affect the treatment efficacy. So acupuncturists should pay attention to elicit *Deqi *and control its state, which is also the key point in modern research on the therapeutic implications of *Deqi*. 
